# Quantitative Structure Inter-Activity Relationship (QS*In*AR). Cytotoxicity Study of Some Hemisynthetic and Isolated Natural Steroids and Precursors on Human Fibrosarcoma Cells HT1080

**DOI:** 10.3390/molecules16086603

**Published:** 2011-08-05

**Authors:** Mihai V. Putz, Marius Lazea, Louis P. Sandjo

**Affiliations:** 1 Laboratory of Computational and Structural Physical Chemistry, Chemistry Department, West University of Timişoara, Pestalozzi Street No.16, Timişoara, RO-300115, Romania; 2 Department of Organic Chemistry, University of Yaoundé 1 P.O. Box 812 Yaoundé, Cameroon

**Keywords:** steroids and triterpenes, organic synthesis, HT1080 cancer cell lines, cytotoxicity activity, Hansch indices, mono and multi-linear correlations

## Abstract

Combined experimental and quantitative structure inter-activity relationship (QSIAR) computation methods were advanced in order to establish the structural and mechanistic influences that steroids and triterpenes, either as newly synthesized or naturally isolated products, have on human HT1080 mammalian cancer cells. The main Hansch structural indicators such as hydrophobicity (LogP), polarizability (POL) and total energy (Etot) were considered and both the structure-projected as well as globally computed correlations were reported; while the inter-activity correlation of the global activity with those projected on structural information was revealed as equal to the direct structural-activity one for the trial sets of compounds, the prediction for the testing set of molecules reported even superior performances respecting those characteristic for the calibration set, validating therefore the present QSInAR models; accordingly, it follows that the LogP carries the most part of the cytotoxic signal, while POL has little influence on inhibiting tumor growth—A complementary behavior with their earlier known influence on genotoxic carcinogenesis. Regarding the newly hemisynthetic compounds it was found that stigmasta-4,22-dien-3-one is not adapted for cell membrane diffusion; it is recommended that aminocinnamyl chlorohydrate be further modified in order to acquire better steric influence, while aminocinnamyl-2,3,4,6-*O*-tétraacétyl-α-D-glucopyranoside was identified as being inhibited in the tumor cell by other molecular mechanisms–here not revealed–although it has a moderate-high anti-cancer structurally predicted activity.

## 1. Introduction

Steroids and their triterpene precursors are both natural product classes widespread in plants and possessing a broad spectrum of biological activities. This family of secondary metabolites is still a research target since many of its compounds such as betulinic, oleanolic and ursolic acids have been reported as antitumor agents [1], while stigmasterol which is one of the biosynthetic descents of triterpenes have significant anticancer activity [2], analgesic [3] and hypoglycemic effects [4]. Moreover, many previous studies have shown that some phytocomponents related to cholesterol presented antimalarial activities [5], or induced apoptosis [6]. However, most of steroids presented in the literature as cytotoxic compounds are polyhydroxylated or contain a α,β-unsaturated ketone function [7,8,9,10]. Besides, these terpenoids caused cell death when they are presented as sulfate [11] or quaternary ammonium salts [12].

On the other side, apart of (hemi)synthesis and isolation of natural products the identification of their anti-cancer molecular mechanism represents one of the main current research concerns [13,14]. In this regard, chemoinformatics in general [15] and the recent spectral and residual variants of quantitative structure-activity relationship (QSAR) in particular [16,17], may offer a reliable *in silico* (or computational) analysis for establishing whether a series of compounds may indeed constitute a fruitful direction of clinical investigations. Basically, the cytotoxic molecular mechanism may be decomposed into three elementary steps: The compound effect on membrane structure, on the signal transduction pathways, and on sterical induction signal towards the cancer cells; these effects can be structurally modeled by the so called Hansch indices’ [18] such as hydrophobicity (LogP), polarizability (POL) and optimized total energy (Etot) influence on the globally observed cytotoxic activity (A), respectively. However, having to account on the cumulative effect of each of these steps in producing the recorded output of programmed cancer cell death, a sort of inter-activity QSAR should be investigated, *i.e.*, by correlating the overall cytotoxic activity with its projection on structurally-based independent activities for the above Hansch indicators. Such a study is to be in the present report unfolded upon a congeneric class of steroids and triterpenes, among which some new hemisynthetic ones were also included.

## 2. Chemistry and Cytotoxicity Assay

**Scheme 1:**
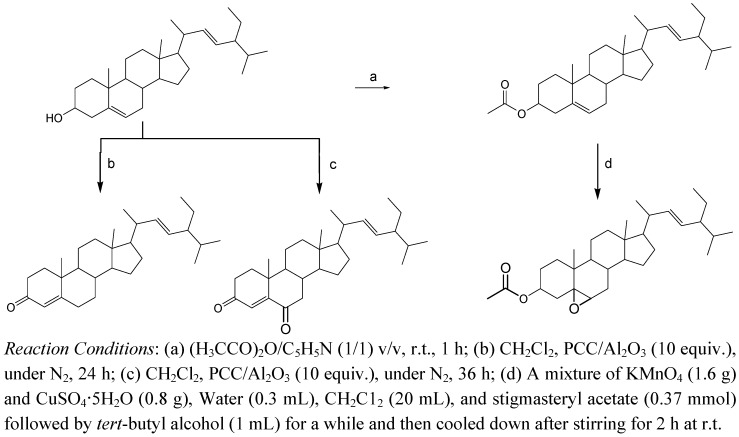
Δ^5^ epoxidation of steroids.

**Scheme 2:**
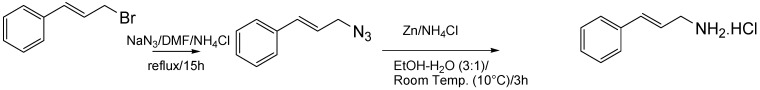
Preparation of aminocinnamyl chlorohydrate.

**Scheme 3:**
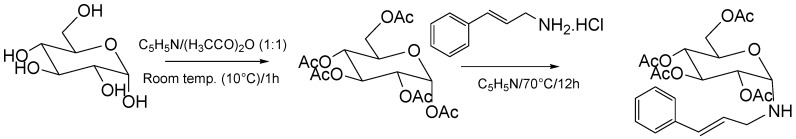
α-Glucosylation.

With the aim to discovering new bioactive components, chemical transformations of stigmasterol were carried out following the route reaction described by Syamala *et al.* [19] (Scheme 1) while the oxidation reaction with pyridinium chlorochromate (PCC) presented was performed as reported by Li *et al.* [20]. For instance, the preparation of aminocinnamyl chlorohydrate was done following the reaction of Scheme 2 [21], while the α-glucosylation reaction is new and presented in Scheme 3.

From the compounds of Table 1, the semisynthetic three pentacyclic triterpenes [lupeol (**1**), oleanolic acid (**2**) and maslinic acid (**10**)] and two steroids (stigmasterol and sitosterol 3-*O*-β-D-gluco-pyranoside) isolated from the stems of cultivated *Triumfetta cordifolia* (Tiliaceae) were tested against HT1080 cancer cell lines, together with as well as the hemisynthetic steroids **3/G3**, **4/G4** and **18** andtwo cinnamyl derivatives **5/G5**, **11/G11**.

These cells are able to undergo physiological cell death, a process termed apoptosis [22], and are sensitive to a wide variety of cytotoxic drugs such as etoposide, cisplatin, staurosporine or TNF-α. In order to avoid a putative toxicity of the solvents, the maximum used volume of the compound solution never exceeded 1/100 of the total volume of culture medium. Controls with analogous concentrations of solvents were always carried out in parallel to check their lack of toxicity. Six compounds were prepared at 100 mM allowing testing concentrations as high as 1 mM, whereas the other five compounds showed lower solubility, consequently eliminating the possibility to test such concentrations.

**Table 1:** Some hemisynthetic and isolated natural products along their IC_50_ cytotoxic activity on human fribosarcoma cell HT1080 and the computed [23] QSAR properties for the Hansch parameters, *i.e.*, hydrophobicity, polarizability and the (PM3) total energy.

No./G/NG *	Name (*Hemisynthetic*, *if any*)	Chemical Formula	Structure	IC_50_ (mM) (observed hours) [A = log_10_(1/IC_50_)]	QSAR properties		
					LogP	Pol [Å^3^]	−Etot (kcal/mol)
1/G1	lup-20(29)-en-3β-ol (Lupeol)	C_30_H_50_O	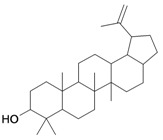	1 (48) [0.000]	8.03	52.4	106582.9141
2/G2	3β-hydroxyolean-12-en-28-oic acid (Oleanolic acid)	C_30_H_48_O_3_	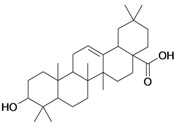	0.7 (48) [0.155]	7.32	53.12	119358.2422
3/G3	3-*O*-acetyl-5,6 epoxystigmast-22-ene-3-ol (*Hemisynthetic*)	C_31_H_50_O_3_	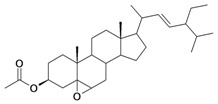	0.54 (48) [0.268]	6.30	54.96	122840.5859
4/G4	Stigmasta-4,22-dien-3-one (*Hemisynthetic*)	C_29_H_46_O	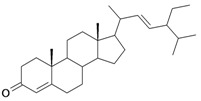	0.3 [0.523]	8.19	50.60	102422.1563
5/G5	Chlorhydrate of Aminocinnamyl (*Hemisynthetic*)	C_9_H_12_ClN	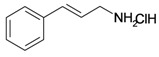	0.1 (48) [1.000]	2.82	19.03	39170.58984
6/G6	Acovenosigenin A or1β,3β,14β-trihydroxycard-20(22)-enolide	C_23_H_34_O_5_	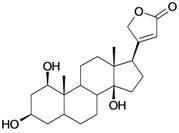	0.0012 [2.921]	2.07	41.55	108835.2969
7/G7	Periplogenin or 3β,5β,14β-trihydroxycard-20(22)-enolide	C_23_H_34_O_5_	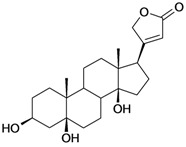	0.00081 [4.092]	1.89	41.55	108837.7656
8/G8	Periplogenin or 3-*O*-β-D-glucopyranosyl-5β,14β-dihydroxycard-20(22)-enolide	C_29_H_44_O_10_	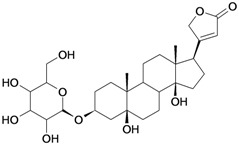	0.00016 [3.796]	0.67	54.97	162652.2813
9/G9	17α-H-digitoxigenin or 3β,14β-dihydroxycard-20(22)-enolide	C_23_H_34_O_4_	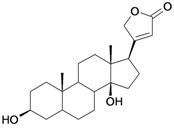	0.0015 [2.823]	3.04	40.91	102065.0234
10/G10	1α,2β-dihydroxyolean-12-en-28-oic acid (Maslinic acid)	C_30_H_48_O_4_	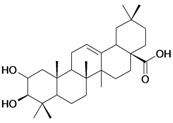	0.075 (48) [1.125]	6.55	53.76	126133.4297
11/G11	Aminocinnamyl-2,3,4,6 -*O*-tetraacetyl- α-D-glucopyranoside (*Hemisynthetic*)	C_23_H_29_NO_9_	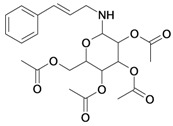	0.88 (48) [0.056]	1.13	45.54	137839.3281
12/NG1	Ginsenoside 20(*S*)-protopanaxadiol or 3β,12β,20S-trihydroxydammar-24-ene	C_30_H_52_O_3_	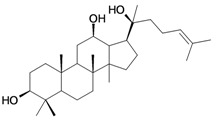	0.07678 [1.115]	6.29	54.45	120871.4531
13/NG2	Acovenosigenin A digitoxoside or 3-*O*-β-D-digitoxopyranosyl-1β,14β-dihydroxycard-20(22)-enolide	C_29_H_44_O_8_	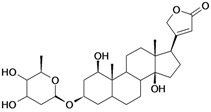	0.0011 [2.958]	2.25	53.70	149114.3281
14/NG3	Periplogenin Digitoxoside or 3-*O*-β-D-digitoxopyranosyl-5β,14β-dihydroxycard-20(22)-enolide	C_29_H_44_O_8_	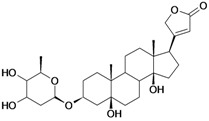	0.000093 [4.032]	2.07	53.70	149112.1719
15/NG4	17α-H-periplogenin-3-O-β-*D*-cymaroside or 3-*O*-β-D-cymarosyl-5β,14β-dihydroxycard-20(22)-enolide	C_30_H_46_O_8_	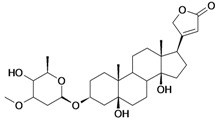	0.000096 [4.018]	2.35	55.53	152546.9375
16	Tormentic acid	C_30_H_48_O_5_	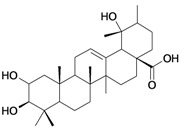	Toxicity < 50% for max 100 µM dose			
17	stigmasterol	C_29_H_48_O	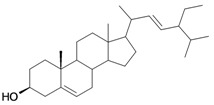	Toxicity < 50% for max 500 µM dose			
18	Stigmasta-4,22-dien-3,6-dione (*Hemisynthetic*)	C_29_H_44_O_2_	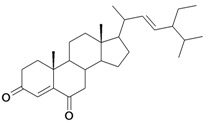	Toxicity < 50% for max 10 µM dose			
19	3-*O*-β-D-glucopyranoside of sitosterol	C_35_H_60_O_6_	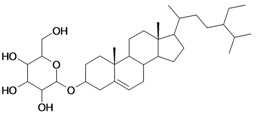	Toxicity < 50% for max 100 µM dose			

**Figure 1:**
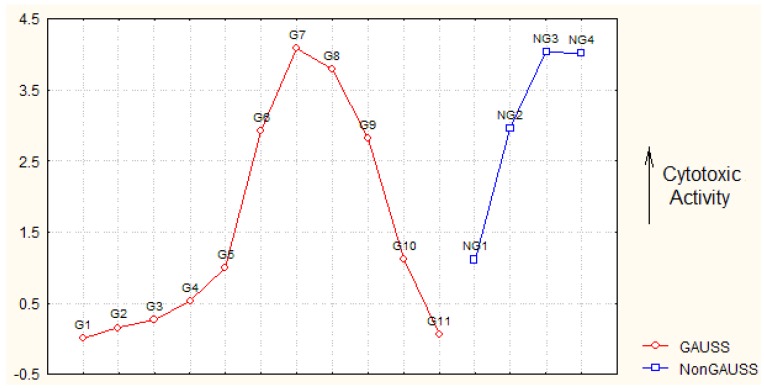
Representation of the cytotoxic activities A = log_10_(1/IC_50_) for the molecules of Table 1 on Gaussian (G) and Non-Gaussian (NG) curves as corresponding to the trial and testing sets of compounds in Table 1, respectively.

* Of Gaussian (G) or Non-Gaussian (NG) type, see Figure 1.

Among these compounds (see Experimental section), four of them (molecules **16–19** of Table 1) induced less that 50% cell death after 48 h of treatment at maximum concentrations, rending impossible the determination of an IC_50_: This was the case for tormentic acid (C_max_ = 100 μM), stigmasterol (C_max_ = 500 μM), stigmasta-4,22-dien-3,6-dione (C_max_ = 10 μM) and 3-*O*-β-D-glucopyranoside of sitosterol (C_max_ = 100 μM).

The poor solubility of some of these compounds, such as stigmasta-4,22-dien-3,6-dione (C_max_ = 10 μM) was undoubtedly a great disadvantage to test their putative cytotoxicity. However, maslinic acid induced 50% of cell death after 48 h of treatment (IC_50_) at 0.075 mM, thus showing a powerful and specific cytotoxic effect. Among the six compounds which were prepared at 100 mM, it has been possible to determine an IC_50_ value for each of them, from the most to the less efficient (Table 1): Aminocinnamyl chlorohydrate (IC_50_ = 0.1 mM), stigmasta-4,22-dien-3-one (IC_50_ = 0.3 mM), 3-*O*-acetyl-5,6-epoxystigmast-22-ene-3-ol (IC_50_ = 0.54 mM), oleanolic acid (IC_50_ = 0.7 mM), amino-cinnamyl-2,3,4,6-*O*-tetraacetyl-α-D-glucopyranoside (IC_50_ = 0.88 mM) and lupeol (IC_50_ = 1 mM). Thus, three of the tested compounds appear to have both good solubility and high specificity (IC_50_ ≤ 0.3 mM) to induce an efficient cellular toxicity in fibrosarcoma cells: Maslinic acid, aminocinnamyl chlorohydrate and stigmasta-4,22-dien-3-one. It is probable for the other compounds that the lack of one or both of these properties would be a disadvantage to trigger an effective *in vivo* cytotoxicity.

## 3. Inter-Activity Models and Discussion

The analyzed molecules of Table 1 are grouped on Gaussian (G) and Non-Gaussian (NG) sets, according with their activity ordering, see Figure 1; they will correspond with the trial/calibration and the test/predicting QSAR series, respectively.

### 3.1. Calibration Quantitative Structure Inter-Activity Relationship (QSInAR)

For Gaussian (trial) compounds one has the basic QSAR analysis: The correlation equations of the recorded activities with the Hansch parameters of Table 1, along the statistical indicators as the Pearson correlation (R) and the standard error of estimation (SEE), either as individual structure projected parameter-activity expressions:

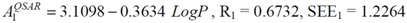
(1)

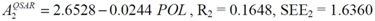
(2)

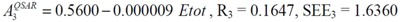
(3)
as well as the global structure-activity multi-regression equation:

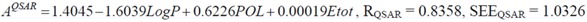
(4)


Equation (4) represents the ordinary QSAR predictive equation; note that the small correlation of the total energy in Equation (3) and with the very small coefficient in (4) is the indication that while the physicochemical properties of a small molecule (like steroids are) are definitely important (e.g., for transport), the adverse effect may stem from the interaction of such compound with a macromolecule (as the actual endpoints are very complex).

Such an observation further suggests that another sort of total-energy related quantity should be considered in multi-regressions; as such, not the energy itself but its projected activity given by Equation (3) is to be next considered; while doing the same with the other parameters too, one arrives to provide the so called inter-activity correlation or the quantitative structure-inter-activity relationship (QSInAR), that is correlating the activity of the Gaussian molecules of Table 1 with the projected activities on the Hansch structural parameters of Equations (1)–(3), and reported in Table 2.

Accordingly, the obtained multi-linear inter-activity regression of global activities of Table 1 with projected activities of Table 2 is unfolded for the training set with the form:

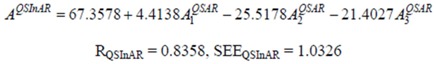
(5)


It is immediate that Equation (5) displays two major features, namely:

- It does not reproduce Equation (4) in regards to the computed coefficients of the structurally-projected activities, while
- Displaying equal correlation respecting the direct structural-parameters-activity correlation of Equation (4).

The quantitative structure inter-activity relationship (QSInAR) may thus be used for further modeling and prediction on equal footing with the original QSAR modeling. The external prediction test is unfolded next.

**Table 2:** The activities for the Gaussian (trial) molecules of Table 1 as computed by Equations (1)–(3).

Index	A_1_^QSAR^	A_2_^QSAR^	A_3_^QSAR^
**G1**	0.191	1.372	1.473
**G2**	0.449	1.355	1.583
***G3***	***0.820***	***1.310***	***1.613***
***G4***	***0.133***	***1.416***	***1.438***
***G5***	***2.085***	***2.188***	***0.90***
**G6**	2.358	1.638	1.493
**G7**	2.423	1.638	1.493
**G8**	2.866	1.310	1.954
**G9**	2.005	1.653	1.435
**G10**	0.729	1.339	1.641
***G11***	***2.699***	***1.540***	***1.741***

### 3.2. Predictive QSInAR

However, in order to validate the present inter-activity approach, both QSAR and QSInAR-tests are now performed for the Non-Gaussian/NG (test) molecules of Table 1 by employing the Gaussian correlation Equations (4) and (5) to produce the data reported in Table 3, respectively. Finally, the correlations between the observed activities and those structurally either as ordinary QSAR or by actual QSInAR prediction analysis are presented in Equations (6) and (7) by employing the data of Table 1 and Table 3 for the Non-Gaussian/NG molecules, respectively as:

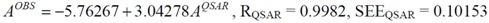
(6)

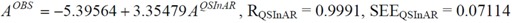
(7)


**Figure 2:**
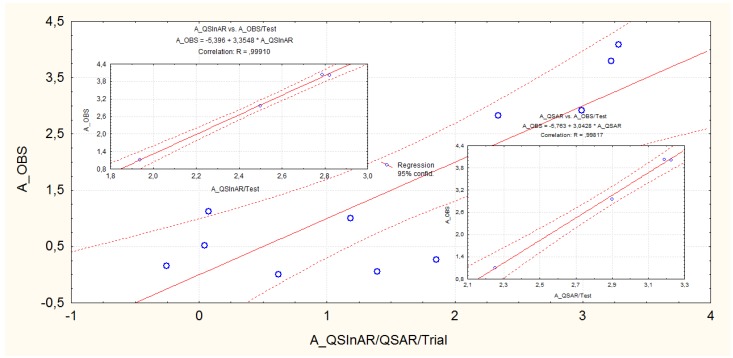
Scatter-plots representations of the observed *vs.* predicted correlations for the ordinary QSAR and inter-activity correlation QSInAR for both trial (Gaussian/G), the central figure, and for the test (Non-Gaussian/NG) sets, the two diagonal extreme insets, according with the regression Equations (4), (5) and (6), (7), respectively. Note that for the trial molecules the QSAR and QSInAR correlations are identically, see Equations (4) and (5), while for the test molecules the data of Table 3 were used.

The QSInAR comparative statistics between trial and test series of Table 1 and of Figure 1 are graphically represented in Figure 2 based on the basic Equations (5) and (7), respectively; the improvement of correlation for the test vs. trial correlation is evident, while being QSInAR superior with respect to ordinary QSAR analysis.

**Table 3:** The activities for the Non-Gaussian/NG (or test) molecules of Table 1 as computed upon Equations (1)–(3), alongside the predicted and computed activities as prescribed by Equations (4) and (5), respectively.

Index	A_1_^QSAR^	A_2_^QSAR^	A_3_^QSAR^	A^QSAR^	A^QSInAR^
**NG1**	0.824	1.324	1.648	2.251	1.938
**NG2**	2.292	1.343	1.902	2.898	2.500
**NG3**	2.358	1.343	1.902	3.187	2.787
**NG4**	2.256	1.298	1.933	3.224	2.822

Overall, one can draw the main features revealed by the present analysis:

- The tested correlation performances are notably superior to those recorded for the calibration set for both QSAR and QSInAR frameworks; this way giving more confidence to the present models as reliable predictive activity tools for future designed steroids and triterpenes;
- Although the interactivity QSInAR model is equal with the direct QSAR one in the trial-correlation set, see Equations (4) and (5), it is slightly superior for the externally tested one, see Equations (6) and (7);
- The consecrated triplet of Hansch parameters (LogP, POL and Etot) is sufficiently powerful to provide significant and reliable correlations of the structure with the measured activities of steroids and triterpenes, yet their projected activities are even more efficient in prediction when considered together in the inter-activity correlations;
- For hemisynthetic compounds **4** one record in Table 2 shows that it reaches the minimum (LogP) projected computed (QSAR) activities in the Gaussian series; the extreme values are also seen for the hemisynthetic compound **5** for POL and Etot projected (QSAR) computed maximum and minimum activities, respectively; this means that these compounds are not predicted as active through the membrane transgression (compounds **4**) and by the steric interaction (compound **5**) but only by signal transduction (compound 5);
- Instead, the hemisynthetic compound **11**, that was globally observed with the lowest cytotoxicity activity in Table 1, it is predicted as having moderate anti-cancer activity especially by membrane diffusion, but also through polarizability and steric effects on tumoral cells; this means that this compound is especially adapted for membrane transduction, while being afterwards somehow inhibited by other (by this approach not revealed) molecular mechanisms during its interaction with tumor cell; further studies may improve upon replacing the total energy indicator, that is strongly depending on the size of the molecule and the number of hydrogen-bonding partner it features, with a key energy information relaying on the binding-site models (atomistic, quasi-atomistic, virtual) which might serve to estimate the strength of the interaction;
- Polarizability has little influence on the recorded activity, see eq. (2), this way eliminating, in principle, the electrophilic mechanism specific to modeling genotoxic carcinogenity [24]; this is naturally since, in fact, the present study reveals the cytotoxic series that due to their induced cancer cell apoptosis acts contrarily to those producing cancer.

## 4. Experimental

### 4.1. General

A Bruker DRX-400 MHz spectrometer was used for 1D ^1^H-NMR studies. Melting points were measured in a Stuart Scientific Melting Point apparatus SMP_3_ and they are uncorrected. The matrix MALDI-TOFMS were a saturated 2,5-dihydroxybenzoic acid (2,5-DHB) solution in 50% methyl alcohol, 0.1% trifluoroacetic acid (TFA) and 2,5-dihydroxybenzoic acid (2,5-DHB, 1 M) solution in 50% acetonitrile / 50% ultrapure water / 0.1% trifluoroacetic acid (TFA), respectively. All the depositions were made using the dried-droplet method. MALDI-TOFMS measurements were possible using a Bruker Reflex IV time-of-flight mass spectrometer (TOFMS) (Bruker-Daltonics, Bremen, Germany) equipped with the SCOUT 384 probe ion source, using a nitrogen pulsed laser (337 nm, model VSD-337ND, Laser Science Inc., Boston, MA, USA) with energy output of 400 μJ/pulse. Vacuum column chromatography, column chromatography and thin layer chromatography were performed respectively on silica gel 60H (particle size 90% < 45µm), 200–300 mesh silica gel, and silica gel GF254.

### 4.2. Plant Material

*T. cordifolia* A. Rich was collected from the central Yaoundé province of Cameroon and a specimen (N° 12830SRF Cam) has been deposited in the National Herbarium of Yaoundé, Cameroon.

### 4.3. Isolation

Dried stems of wild species of *Triumfetta cordifolia* A. Rich (2.55 Kg) were cut into small pieces and powdered. Powder (250 g) was extracted with methanol by heating for 8 h. Methanol extract (60 g) was concentrated under vacuum and partitioned between water and ethyl acetate. Both phases were concentrated under vacuum to give 35 g of organic phase and 24 g of aqueous extract. The ethyl acetate extract was subjected to flash chromatography (silica gel, cyclohexane, cyclohexane-EtOAc 3:1-1:1-1:3, EtOAc, in order of increasing polarity) yielding five fractions. The cyclohexane-EtOAc (3:1) fraction was purified by column chromatography with different mixtures of cyclohexane-EtOAc yielding 217 fractions. The fractions 22–32 eluted with the mixture cyclohexane-EtOAc (9:1) yielded 32.0 mg of lupeol (**1**). The ones (40–52) eluted with the mixture cyclohexane-EtOAc (17:3) yielded 45 mg of oleanolic acid (**2**). The flash chromatography fraction from mixture of cyclohexane-EtOAc (1:1) yielded 125.0 mg of stigmasterol (**17)** in the mixture cyclohexane-EtOAc (17:3), 2α-Hydroxyoleanolic acid (32.5 mg) was obtained from mixture of cyclohexane-EtOAc (3:1). The fraction obtained from mixture of cyclohexane-EtOAc (1:3) was eluted with CH_2_Cl_2_-MeOH mixtures of increasing polarity. The fractions 54–59 eluted with the 19:1 CH_2_Cl_2_-MeOH mixture gave the β*-*D-glucopyranoside of β-sitosterol (11.0 mg). The dried aqueous partition was extracted with acetone yielding 20 mg of extract. This was eluted with a CH_2_Cl_2_-MeOH mixture of increasing polarity to yield 114 fractions. Fractions 74–80 eluted with 37:3 mixture of CH_2_Cl_2_-MeOH yielded tormentic acid (30 mg).

### 4.4. Cellular Viability

Given that solvents are often toxic to mammalian cells, the compounds were dissolved at high concentration (100 mM). When the solubility did not allow us to reach 100 mM, we tried to prepare the most concentrated solutions possible. Lupeol, oleanolic acid, 3-*O*-acetyl-5,6-epoxystigmast-22-ene-3-ol, stigmasta-4,22-dien-3-one and aminocinnamyl-2,3,4,6-*O*-tetraacetyl-β-D-glucopyranoside were prepared at 100 mM in THF. Aminocinnamyl chlorohydrate was prepared at 100 mM in DMSO. Stigmasterol and stigmasta-4,22-dien-3,6-dione were prepared at 50 mM and 1 mM, respectively, in THF. Finally, maslinic acid and both tormentic acid and the 3-*O*-β-D-glucopyranoside of sistosterol were prepared in DMSO at 50 mM and 10 mM, respectively.

The human HT1080 fibrosarcoma adherent cell line was cultured at 37 °C in a humidified atmosphere containing 5% CO_2_ in Dulbecco’s modified Eagle’s medium (DMEM/F12) supplemented with 10% fetal bovine serum together with penicillin (100 μg/mL), streptomycin (100 U/mL) and glutamax (1% v/v) from Invitrogen. The cells were seeded in 12 well plates (5 × 10^4^ cells/well). After 24 h, the medium was replaced in each well by 1 mL of complete medium with the appropriate concentrations of the tested drugs. Corresponding controls with analogous concentrations of solvent were carried out in parallel. Cells were then incubated for 48 h and cellular viability was determined by flow cytometric analysis. Since type I (apoptosis), type II (autophagy) and type III (necrosis) cell death trigger permeabilization of mitochondria [25], overall cell death was then determined with the cationic lipophilic DiOC_6_(3) dye (Invitrogen) which specifically probes mitochondrial membrane potential (ΔΨm) [26]. A decrease in forward light scattering (FSC) was also checked to confirm the cell death process [27]. After drug treatment, the media from each well were kept in centrifuge tubes. The adherent cells were detached using trypsine, pooled with the corresponding media, centrifuged and resuspended in complete medium. Cells were then loaded with 100 nM DiOC_6_ (3) and incubated for 30 min at 37 °C. Finally, cells were kept on ice prior flow cytometric analysis. Flow cytometric measurements were performed using a XL3C flow cytometer (Beckman-Coulter). Fluorescence was induced by the blue line of an argon ion laser (488 nm) at 15 mW. Green fluorescence of DiOC_6_ (3) was collected with a 525-nm band pass filter. Analyses were performed on 10^4^ cells.

### 4.5. Compounds Chosen from the Literature

The compound **12** was obtained from the Research Institute of Luye Pharmacy, Yantai, China and its cytotoxic activity on human fibrosarcoma was tested [28]. Its melting point was measured on the Fisher micromelting point apparatus (hot-stage) and recorded uncorrected [29]. The compounds **6–9**, and **13–15** were isolated and identified from *Streptocaulon juventas* [30] and their cytotoxic properties investigated [31,32].

## 5. Conclusions

Since nowadays there is increasing interest in public health through various environmental interactions, the design of chemical compounds with little toxic effects on organisms or with anti-cancer effects remains at the forefront of actual molecular research either by experimental synthesis and computational modeling. In this context, the present work sought to combine both aspects (experimental and computational) in reporting new molecules in a series with pre-defined cytotoxicity activities. As such, the chemical synthesis of two new stigmast-en-ol/one (compounds **3** and **4** of Table 1) and aminocinnamyl- (compounds **5** and **11** of Table 1) derivatives were described and their cytotoxicity influence on human fibrosarcoma cells HT1080 analyzed by means of structural projected activities within the so called quantitative structure inter-activity relationship (QSIAR) modeling. The results are quite relevant, and are summarized below:

- The cytotoxicity of congeneric (both trial and test) series was considered and modeled as being projected on three fundamental Hansch parameters such as LogP, POL and Etot; it was found that the hydrophobicity (LogP) is the main mechanism of interaction, while the polarizability (POL) has little influence on anti-tumoral mechanism—Rejecting this way the eventual generalization of Miller’s electrophilic theory of carcinogenicity to its reversible evolution though cancer cells’ apoptosis; in fact, while LogP was found to have little influence on carcinogenity [17], it appears here as having the main influence; one may conclude therefore that indeed the carcinogenity (formation of cancer cells) and the cytotoxicity (death of cancer cells) are complementary and not symmetrical with respect to QSAR analysis;
- The presented inter-activity QSInAR correlation equations show overall slightly better statistical prediction with respect to the custom QSAR counterpart, while providing equal performance for our trial set of newly hemisynthesyzed and isolated natural steroids and precursors triterpenes; this behavior assures the present reliable QSInAR analysis and the conclusions that result from it, namely:
  ○ The newly hemisynthesized compounds are not all well suited to accounting for cytotoxicity of HT1080 cells, while their anti-cancer action is more relevant through the projection on some particular stages of molecular mechanism of ligand-receptor interaction, as following:
  ○ The compound stigmasta-4,22-dien-3-one (compound **4** in Table 1) is particularly not adapted for membrane transduction and therefore should be omitted from further clinical studies;
  ○ The compound aminocinnamyl chlorohydrate (compound **5** in Table 1) is less effective in tumoral apoptosis by steric signals but potentially active by polarization and therefore it should be further developed into another derivatives with improved structural projected activities;
  ○ Aminocinnamyl-2,3,4,6-*O*-tetraacetyl-α-D-glucopyranoside (compound **11** in Table 1) although it recorded the lowest observed overall molecular activity it is predicted to have moderately high cyt-activities as projected on all hydrophobic, polarizability and steric influences; such a behavior suggest that it undergoes successive molecular interaction within the cancer cells where some unrevealed inhibition interaction impedes its structurally-projected activities from being globally manifest towards tumor apoptosis.

While the ideal case will be the equal cytotoxicity performances at both local (structurally-projected) and global (observed) activities, one may nevertheless rely on the inter-activity analysis and make further recommendations for new synthesis and molecular interactions and mechanism as far as it provides statistical relevant results for both trial and test congeneric molecules when physicochemical working structural indicators are included. The present work provides the first step in this combined experimental-QSInAR analysis and there is hope that it will be followed by other similar dedicated studies.
